# Deep Color Transfer for Color-Plus-Mono Dual Cameras

**DOI:** 10.3390/s20092743

**Published:** 2020-05-11

**Authors:** Hae Woong Jang, Yong Ju Jung

**Affiliations:** College of Information Technology Convergence, Gachon University, Seongnam 1342, Korea; leap1568@gmail.com

**Keywords:** dual camera, color transfer, convolutional neural network (CNN), low-light enhancement

## Abstract

A few approaches have studied image fusion using color-plus-mono dual cameras to improve the image quality in low-light shooting. Among them, the color transfer approach, which transfers the color information of a color image to a mono image, is considered to be promising for obtaining improved images with less noise and more detail. However, the color transfer algorithms rely heavily on appropriate color hints from a given color image. Unreliable color hints caused by errors in stereo matching of a color-plus-mono image pair can generate various visual artifacts in the final fused image. This study proposes a novel color transfer method that seeks reliable color hints from a color image and colorizes a corresponding mono image with reliable color hints that are based on a deep learning model. Specifically, a color-hint-based mask generation algorithm is developed to obtain reliable color hints. It removes unreliable color pixels using a reliability map computed by the binocular just-noticeable-difference model. In addition, a deep colorization network that utilizes structural information is proposed for solving the color bleeding artifact problem. The experimental results demonstrate that the proposed method provides better results than the existing image fusion algorithms for dual cameras.

## 1. Introduction

In low-light environments, an image that is captured by a single RGB color camera using a Bayer color filter array (CFA) usually suffers from high noise and low detail information due to low-quantum efficiency. Various studies have been conducted to enhance images captured under low-light conditions to overcome these shortfalls of Bayer color cameras. Among them, dual camera approaches that utilize an additional monochrome camera without CFA have shown promising results in low-light photography. It is known that a monochrome camera can obtain images with less noise and improved detail visibility because of the absence of a color filter array [1]. An image fusion technique is used to apply these features of a monochrome camera to a corresponding noisy color image. The image fusion technique for a dual camera approach includes two processing steps: per-pixel registration and two image combination. In general, the physical positions of color and mono cameras are slightly separated in the horizontal direction. Thus, the disparity of a color- and mono-image pair should be computed by a stereo matching algorithm and compensated for per-pixel registration. Subsequently, the color- and mono-image pair can be combined by appropriate fusion rules to obtain a visually pleasurable output image.

However, the image fusion using a color-plus-mono dual camera might not always produce visually plausible results due to inaccurate per-pixel registration [2]. In recent years, a few studies have investigated per-pixel registration for image fusion [3,4,5,6]. However, multi-spectral stereo-image pairs that are captured by color-plus-mono dual cameras make stereo matching more difficult [4]. Therefore, inaccurate stereo matching can produce erroneous per-pixel registration. If color-mono image fusion is performed after inaccurate per-pixel registration with disparity errors, visual artifacts, such as structural distortions and false colors, are generated in the fused image result [2]. Therefore, a robust fusion solution to erroneous disparity estimations is essential.

In general, two approaches are used to fuse color- and mono-image pairs in a color-plus-mono dual camera: (1) color transfer [4,5,6,7,8,9,10,11,12,13,14] and (2) detail transfer [15].

The first approach is to colorize the mono image by using the color information of the corresponding color image (in this study, this approach is called “color transfer”). This approach preserves the mono image, as it is in the final fused image. Therefore, all detailed information of the mono image can be preserved. The color transfer method can be implemented through reference-based colorization or hint-based colorization.

Reference-based colorization (also known as exemplar-based colorization) is a method that finds appropriate color information on a reference image and propagates it in a target image. Traditional reference-based colorization algorithms [7,8,9] typically use hand-crafted features to search the best matching between the reference and target images. In a recent study, He et al. [10,11] proposed deep learning-based methods for reference-based colorization. He’s methods showed better performance than the traditional methods utilizing the hand-crafted feature. Meanwhile, specialized methods have been proposed for the color transfer of color and monochrome cameras [4,5,6]. Jeon et al. [4] proposed a stereo-matching method for the color transfer. Their method also includes a least-square optimization-based colorization algorithm that is based on the colorization technique of Levin et al [12]. 

Dong et al. proposed deep learning-based colorization methods for a color-plus-mono dual camera [5,6]. Dong’s method [5] transfers color information through patch-based matching and deep learning-based coarse-to-fine colorization. In a recent study [6], they used a strategy of the weighted sum of multiple color hints in the scanline of a stereo image pair, rather than only transferring one hint at a position.

Hint-based colorization [12,13] is to propagate the color hints that are given by users for a target image. The hint-based colorization method generally requires user’s scribbles or strokes as color hints. Recently, Zhang et al. [13] proposed a deep learning-based method. Note that the hint-based colorization methods can be applied for the color transfer of color and monochrome cameras if the color hints are obtained by stereo matching for a color- and mono-image pair.

**Figure 1:**
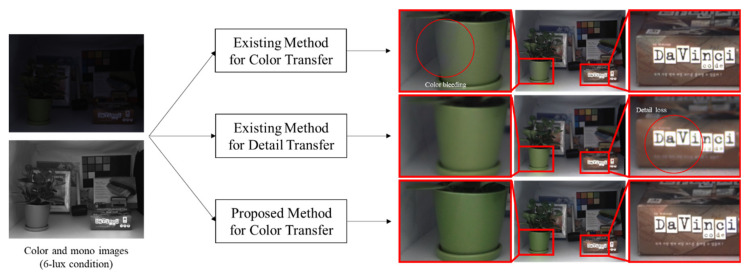
Example of image fusion for color-plus-mono dual camera. The first row shows the result obtained by the previous method of color transfer [6]. The middle row shows the result obtained by the previous method of detail transfer [15]. The last row shows the result obtained by the proposed method.

However, some limitations of the existing colorization methods have to be considered before applying for the image fusion of a color- and mono- image pair for the purpose of low-light image enhancement. Most methods fail to correctly match the color information to the corresponding pixels of the mono image captured in low-light conditions. Additionally, the existing methods cannot handle dominant errors in large occlusion areas. Note that incorrect matches result in unreliable color hints for colorization. Furthermore, the existing methods for colorization [5,6,12,13] often suffer from color bleeding artifacts (i.e. dominant color hints crossing over the edges in some neighboring regions) that cause a negative impact on the quality of the fused images (see Figure 1).

The second approach is to improve the quality of the color image by using the detailed information of the corresponding mono image (in this study, this approach is called “detail transfer”). This approach preserves the color information and hence does not produce any severe false-color artifacts. However, it requires per-pixel registration of the mono image. Unfortunately, the per-pixel registration of mono image is not accurate because of disparity errors. If inaccurate detail information that is caused by disparity errors is transferred into the color image, it can result in visual artifacts [2]. In a recent study [15], Jung proposed a selective detail transfer method that selectively transfers the detailed information based on the degree of disparity error of each pixel. The greater the disparity error, the less detail information is transferred. This concept seems to be promising, and the experimental results demonstrate that visual artifacts in the fused image can be mitigated by selectively transferring detail information. However, this method inevitably leads to a loss of detail information, so that the fused image can be blurred in disparity error regions (see an example in Figure 1).

This study proposes an effective color transfer method that is robust to both disparity estimation errors and color bleeding artifacts. The proposed color transfer method consists of (1) a joint denoising step for color and mono images, (2) a color-hint mask generation step for pruning unreliable color hints while using a disparity reliability map computed based on the binocular just-noticeable-difference (BJND) model, and (3) a deep-learning-based colorization step that performs colorization of the mono image using only reliable color hints. Particularly, the proposed colorization model uses pre-calculated edge information to reduce the color bleeding artifacts that often arise with the color transfer approach. Note that the proposed method also performs a joint denoising for a color and mono image pair. However, the same denoising method from [15] is adopted, as this study does not focus on optimizing the denoising process.

In the experiments, the proposed method is compared with the existing methods through various image datasets. The experimental results reveal that the proposed method can substantially improve image fusion performance. In addition, the comparison experiments are conducted while using a stereo dataset that was constructed from a color-plus-mono dual camera. The results show that the proposed method can provide better visual results than those of state-of-the-art methods for image fusion of color- and mono-image pairs.

Our contributions are summarized, as follows:

(1) We propose a reliable color hint selection algorithm based on human binocular model. Incorrect color hints often occur due to the per-pixel registration errors in low-light and multi-spectral (i.e., color-plus-mono camera setup) environments, which lead to false color artifacts in the final fused image. Hence, in this study, incorrect color hints are explicitly removed while using the reliability map based on human binocular model.
(2) We also propose a hint-based colorization model using edge information that can mitigate color bleeding artifacts. The existing colorization methods suffer from the color bleeding artifacts due to the lack of restrictions for color propagation across the edges on neighboring objects. In this study, we propose a deep colorization network that mitigates the color bleeding artifacts by using edge information as additional guidance for colorization.

The remainder of this paper is organized, as follows. Section 2 describes the proposed color transfer method. In Section 3, experiments and results are presented in order to evaluate the performance of the proposed method. Section 4 draws conclusions.

## 2. Methods

**Figure 2:**
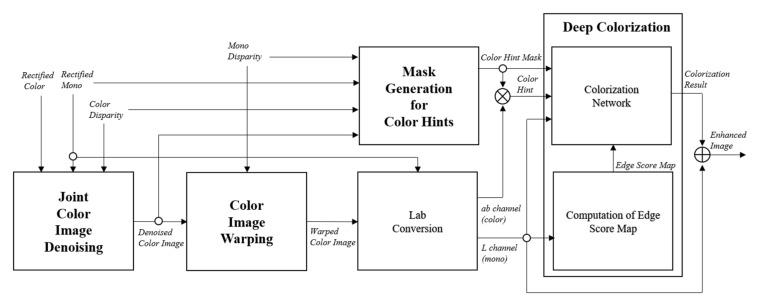
Overall procedure of deep color transfer method for image fusion in color-plus-mono dual camera.

The proposed method is divided into three major parts: denoising, color-hint mask generation, and deep colorization, as shown in Figure 2. In the denoising part, joint image denoising is performed to remove noises from an input color image. In the color-hint mask generation part, a reliability map is first computed to determine reliable color hints. A color-hint mask is then generated using the pixel reliability values to remove unreliable pixels in the warped color image. Finally, the mono image is colorized through a deep colorization network while using the reliable color hints in a Lab color space.

The input of the proposed method is a pair of rectified stereo images (i.e., rectified color and mono images). Note that two image sensors are used to capture the input color and mono images. Therefore, the luminance levels of the color and mono images could be different, particularly in low-light conditions [15]. In this study, the luminance normalization of the two images is performed as a preprocessing step while using the conventional histogram matching method that adjusts the histogram of a color image to match a 64-bin histogram of the reference mono image [16]. In addition, two disparity maps (one each for the color and mono images) are then computed while using a stereo-matching algorithm. In this study, we do not propose a new stereo-matching algorithm for obtaining disparity between color and mono images. Instead, the disparity is calculated while using a publicly available optical flow-based method. Specifically, a TV-L1 optical flow method that is solved with a primal-dual algorithm [17] is used. Here, we use the color image as a reference for stereo matching and estimate the disparity by matching it with the mono image to compute the color disparity.

Note that a color image captured in a low-light environment contains many noises. If colorization is performed on a noisy color image, false color artifacts can occur in the final image. Therefore, denoising the input color image must first be performed for accurate colorization. To this end, we adopt a state-of-the-art joint-denoising method for a color-plus-mono dual camera [15]. A more formal description of the joint-denoising is given in [15].

Per-pixel registration is required to colorize a mono image because of the disparity between color- and mono-images. In this study, color hints for colorizing a mono image are obtained by the backward warping of the color image onto the mono image, as follows:

(1) $$C^{warped}\left(x,y\right)=C^{denoised}\left(x+d_{m},y\right),$$

where *d_m_* denotes the disparity at the pixel (*x*, *y*) in the mono disparity map that is associated with the mono image.

The proposed colorization model is performed in an ab color space. Subsequently, a new Lab image is obtained by concatenating the L and ab channels of the mono and warped color images, respectively. Furthermore, because the ab channels of the warped color image can contain unreliable color-hint pixels, we multiply ab channels by a binary mask obtained, as described in Section 2.1. The color-hint value $\hat{a}\left(x,y\right)$ in the “*a*” color channel to be used in the colorization model is given by:

(2) $$\hat{a}\left(x,y\right)=a\left(x,y\right)\cdot M\left(x,y\right)$$

where $a\left(x,y\right)$ is a warped color channel value and $M\left(x,y\right)$ is the binary mask value that indicates a reliable color hint at a pixel position (*x*, *y*). Likewise, $b\left(x,y\right)$ is the “*b*” color channel value that is obtained using the same mask function. Consequently, Lab input channels, which contain all of the luminance values from the mono image and reliable color hint values from the color image, are given for the colorization step (see Section 2.2 for details). 

### 2.1. Mask Generation for Color Hints

Image-warping errors may occur in the warped color image because of disparity errors. Therefore, using all of the warped color values can generate severe visual artifacts during image colorization. We propose an effective method for binary color-hint mask generation that removes unreliable color hints from the warped color image to address this issue.

**Figure 3:**
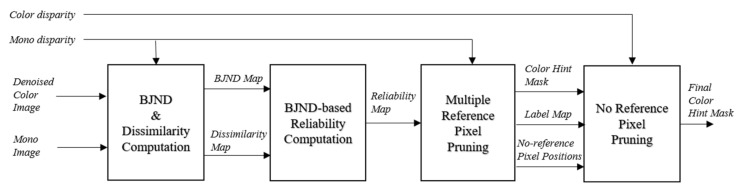
Procedure of the mask generation method for color hints.

**Figure 4:**
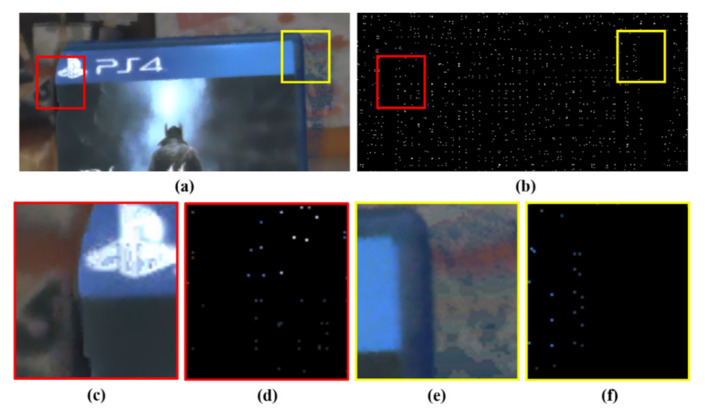
Close-up example of a color-hint image. (**a**) Warped color image with warping errors. (**b**) Binary color-hint mask. (**c**) Close-up view of the red area in (**a**). (**d**) Color hints of the red area in (**b**). (**e**) Close-up view of the yellow area in (**a**). (**f**) Color hints of the yellow area in (**b**).

The proposed mask generation method consists of four parts, as shown in Figure 3. In the first part, BJND and dissimilarity values are calculated for each pixel in the mono image while using the color image and mono disparity. In the second part, a reliability map is obtained using BJND and image dissimilarity. In the third part, unreliable pixels that occur in disparity error regions are removed based on the reliability map. In the fourth part, the remaining unreliable pixels are removed while using a block-based left-right consistency check. Figure 4d,f show that unreliable color hints are pruned. Note that the binary color-hint mask is very sparse. We intend to conservatively prune all possible incorrect color hints as the incorrect color hints can generate visual artifacts in colorization.

#### 2.1.1. BJND-Based Reliability Computation

An appropriate evaluation criterion is necessary to remove incorrect color-hint pixels and retain only reliable color-hint pixels. Incorrect color-hint pixels mainly occur because of misregistration between the corresponding pixels of the color and mono images. Therefore, a basic approach for evaluating misregistration is to calculate the degree of dissimilarity between a pair of color and mono images. The dissimilarity of the stereo image pair can be calculated, as follows:

(3) $$S\left(x,y\right)=\sum_{i=1}^{N_{W}}\sum_{j=1}^{N_{W}}w\left(i,j\right)\left|l_{x,y}^{m}\left(i,j\right)-{\hat{l}}_{\hat{x},\hat{y}}^{c}\left(i,j\right)\right|,$$

where, $l_{x,y}^{m}$ is a local 11 × 11 square window centered at the pixel position of (*x*, *y*) in the input mono image and ${\hat{l}}_{\hat{x},\hat{y}}^{c}$ is a local window centered at the disparity-compensated position (i.e., $\hat{x}=x+d$ and $\hat{y}=y$) in the input color image, in which *d* is the disparity at (*x*, *y*). *w* is an 11 × 11 circular-symmetric Gaussian weighting function with a standard deviation of five samples and is used to mitigate undesirable blocking artifacts [18]. $N_{w}$ is the size of a local window (i.e., $N_{w}$ = 11). Note that the color image is first converted into a grayscale image to calculate Equation (3). In our implementation, a standard conversion method according to the Recommendation of ITU-R BT.601-7 was used [19].

However, only calculating the dissimilarity on two images might be insufficient as an evaluation criterion for pixel-wise reliability. This is because, even when the dissimilarity values of two positions are the same, the misregistered pixels of some regions may be more perceptually noticeable than those of the other regions based on the characteristics of luminance adaptation and contrast masking in a human visual system (HVS) [20].

A just noticeable difference (JND) concept can be utilized to determine whether a color hint is reliable and the artifacts in the final fused image are tolerable to the HVS. In an HVS, the JND is the amount of luminance that must be changed to recognize the difference from the neighbor’s luminance. If the luminance difference between two pixels is less than JND, the HVS cannot recognize the difference. Recently, several studies have been conducted to apply the JND concept to a stereo-image pair [20]. The BJND [20] is a human binocular vision model that represents the visibility threshold for a given stereo-image pair. In [20], it has been experimentally demonstrated that if the difference in pixel luminance between two binocular images is smaller than BJND, a human cannot recognize the difference.

Given a color image, a mono image, and a disparity map associated with the mono image, the BJND at a pixel position (*x*, *y*) is defined, as follows [20]:

(4) $$BJND_{m}\left(x,y\right)=BJND_{m}\left(bg_{c}\left(x+d,y\right),eh_{c}\left(x+d,y\right),n_{c}\left(x+d,y\right)\right),\;=A\left(bg_{c}\left(x+d,y\right),eh_{c}\left(x+d,y\right)\right)\cdot {\left(1-{\left(\frac{n_{c}\left(x+d,y\right)}{A\left(bg_{c}\left(x+d,y\right),eh_{c}\left(x+d,y\right)\right)}\right)}^{\lambda }\right)}^{\frac{1}{\lambda }}$$

where *d* is a disparity value at (*x*, *y*) and 0 ≤ $n_{c}$ ≤ *A*($bg_{c}$, $eh_{c}$). Note that *BJND_m_* for the mono image is a function of the background luminance level *bg_c_*, the edge height *eh_c_*, and the noise amplitude *n_c_* at a pixel position in the color image [15]. *λ* is a parameter that controls the influence of noise in the mono image. In this paper, it is set to 1.25, as mentioned in the previous study [20]. *bg* is the background luminance that is obtained by averaging the luminance of the 5 × 5 window around the corresponding pixel position. *eh* is the edge height obtained by using the Sobel operator at the corresponding pixel position. The function *A* is given by psychophysical experiments, as mentioned in [20].

**Figure 5:**
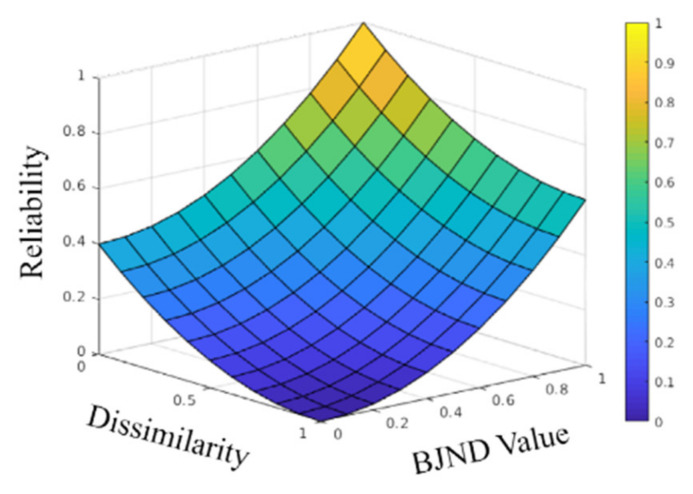
Binocular just-noticeable-difference (BJND)-based reliability function. Reliability values are obtained by dissimilarity and BJND.

In this study, we propose a computation method for a reliability map while using BJND [20] that is used to judge the reliability of a color hint. Specifically, *BJND_m_* and the dissimilarity *S*_m_ for the mono image are calculated through the disparity-compensated color image using the mono disparity, as described in Equations (3) and (4). With the calculated values, the reliability values are computed by:

(5) $$Reliability\left(BJND_{m},S_{m}\right)=\frac{\beta \cdot BJND_{m}{}^{2}+{\left(1-S_{m}\right)}^{2}}{1+\beta },$$

where *β* is a parameter that controls the influence of BJND. In this study, it was set to 1.5 in order to produce visually plausible results in our experiments. Because *BJND_m_* and *S_m_* values are normalized to [0.0, 1.0], the result of the reliability function is fixed to [0.0, 1.0]. Figure 5 shows the reliability function that is modeled by using the BJND and dissimilarity values. As *S_m_* approaches 0 and the BJND value approaches 1, the value of the reliability map increases, as shown in Figure 5. More importantly, despite the same dissimilarity value, the HVS can differently perceive the degree of dissimilarity according to different BJND values [20]. Note that a high BJND value means that the HVS cannot identify the difference in that particular region [20]. Therefore, the higher the BJND of a given pixel, the higher the assigned degree of reliability for that pixel.

**Figure 6:**
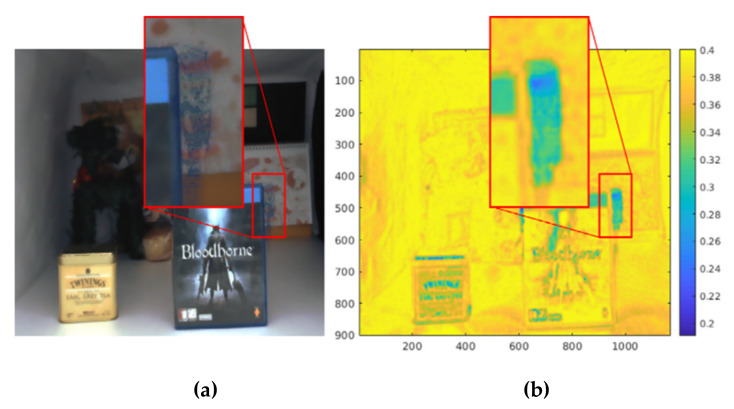
BJND-based reliability map. (**a**) Warped color image. (**b**) Reliability map. The red rectangle indicates a low reliability region.

The final reliability map is obtained by applying an average smoothing filter. The calculated reliability map can be seen in Figure 6. Figure 6b shows that the reliability values in the marked rectangular region are very low, because disparity errors exist in that area.

#### 2.1.2. Multiple Reference Pixel Pruning

**Figure 7:**
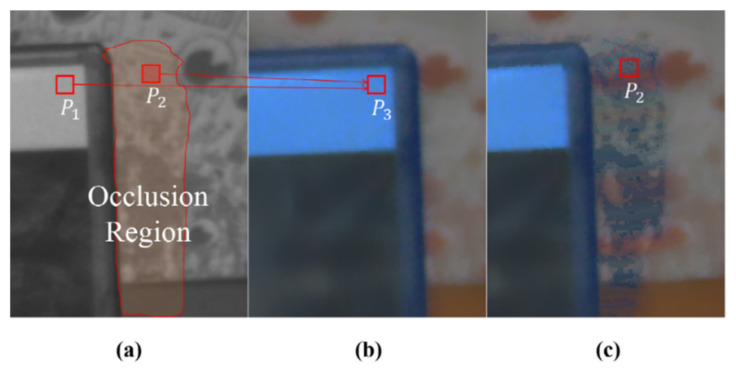
Multiple reference pixel. (**a**) Mono image. (**b**) Color Image. (**c**) Warped color image. Pixels $p_{1}$ and $p_{2}$ in the mono image refer to the same pixel $p_{2}$ in the color image in terms of mono disparity. Note that because the pixel $p_{2}$ belongs to an occlusion area and refers to an incorrect reference $p_{3}$, a visual artifact occurs in (**c**).

Figure 7 shows the tendency of the visual artifact in the warped color image that is caused by disparity errors in the yellow region in Figure 4. This example shows the misregistered result that usually occurs when stereo matching is performed in color occlusion regions (i.e., some foreground object in the color image in Figure 7b occludes the background regions in the corresponding mono image in Figure 7a). Stereo matching algorithms generally assume the uniqueness of matching pixels. It means that a pixel in an image of a stereo image pair must be matched to a certain pixel in the corresponding image. However, this uniqueness constraint is not fully maintained because of occlusion regions. A pixel in the mono image is correctly matched to a pixel in the color image, while the other is mismatched, as shown in Figure 7a. In this case, since the pixel in the occlusion region does not have any matched pixel in the corresponding image, it will be pointed to an incorrectly matched pixel. Thus, this can result in multiple references. Here, note that there is only one correct matching and the other is incorrect matching.

Let us consider that backward warping of the color image is performed on the mono image while using the disparity of the mono image. If the two pixels (e.g., $p_{1}$ and $p_{2}$ in Figure 7) in the mono image refer to the same color pixel (e.g., $p_{3}$ in Figure 7b), then one of them refers to an incorrect color hint, as shown in Figure 7c. In other words, if a color pixel in the color image is referenced multiple times, one is a correct hint and the other is an incorrect hint.

Among the multiple reference pixels (e.g., $p_{1}$ and $p_{2}$), the reliability value of a correct color hint (e.g., $p_{1}$ in this case) will be relatively high. Accordingly, only one pixel with the highest reliability value among the multiple reference pixels is treated as a correct color hint, and the remaining warped color pixels are all pruned as incorrect color hints.

**Figure 8:**
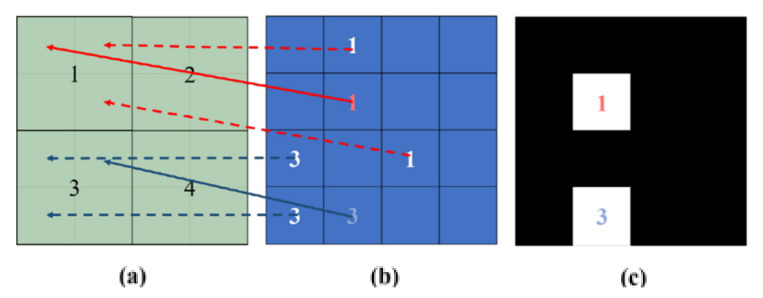
Multiple reference pixel pruning for color-hint mask generation. (**a**) Disparity compensated position in the color image. (**b**) Mono pixels in the mono image. The number in each pixel indicates the block reference number. (**c**) Color-hint mask for the warped color image. In (**c**), white and black pixels represent reliable and unreliable color hints, respectively.

Here, this pruning process is performed on a block basis to prune incorrect hint pixels in a more conservative manner. Figure 8 illustrates the block-based pruning for multiple reference pixels. The warped position is divided into blocks of $N_{B1}\times N_{B1}$ pixels. One label is assigned to each block, as shown in Figure 8a. Subsequently, disparity compensation for all mono pixels is performed. Each pixel in the mono image is assigned a block label that belongs to the compensated position in the color image.

Let *B* be a block label map that indicates the corresponding block number for each pixel of the color image. In addition, let *R*(*p*) be a reliability value at the pixel *p*, as computed in Section 2.1.1. Subsequently, among the pixels with the same label, only one with the highest reliability value is selected as a color hint for the reference block *B_i_*.

(6) $$p_{\max}=\underset{p}{argmax}R(p),\;\mathrm{for}\;\forall p\in \{(x,y)|B(x,y)=B_{i}\},$$

where $B_{i}$ is the $i^{th}\;$ block label. This process is performed for every block number in *B*. Here, the number of color hints depends on $N_{B1}$ because only one color hint exists per block. The higher $N_{B1}$ is, the greater the number of reliable pixels that remain, but the fewer the number of color hints. In our experiment, $N_{B1}$ is experimentally set to 9.

Finally, the locations of the selected color hints (i.e., $P_{max}$) are saved in a binary mask *M*. Note that each pixel value of the binary mask has a value of 0 or 1. A value of 1 means a correct hint pixel (see Figure 8c).

#### 2.1.3. No-Reference Pixel Pruning

**Figure 9:**
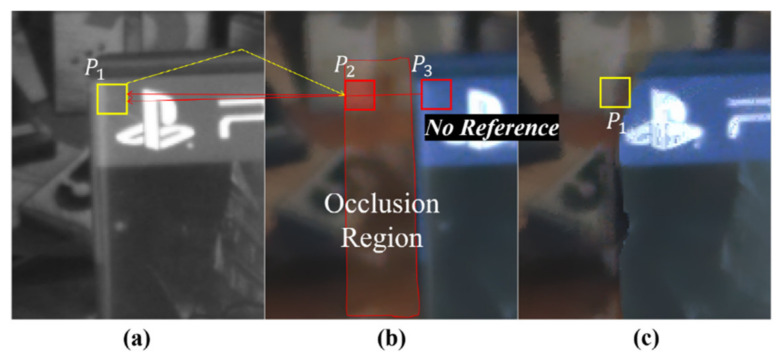
No-reference pixel. (**a**) Mono image. (**b**) Color image. (**c**) Warped color image onto the mono image. In this example, pixel $p_{3}$ in the color image is a correct reference of $p_{1}$ in the mono image in terms of the color disparity. However, $p_{1}$ refers to an incorrect reference pixel, $p_{2}$, in terms of the mono disparity. This misregistration results in severe artifacts in the warped color image in (**c**).

As previously described, examining multiple reference pixels is utilized to remove incorrect color hints in the warped color image. However, incorrect color hints can still remain in the warped color image. Figure 9 shows the tendency of the visual artifacts that are caused by disparity errors in the red region of Figure 4. In terms of the disparity for the color image (i.e., color disparity), both $p_{2}$ and $p_{3}$ refer to $p_{1}$. The correct correspondence of $p_{1}$ in the mono image is $p_{3}$ in the color image, as shown in Figure 9a,b. However, the color pixel $p_{3}$ is not referenced by the mono pixel $p_{1}$ in terms of mono disparity. In this case, severe artifacts occur in the warped color image, as shown in Figure 9c. We should conservatively prune these incorrect color hints because only a few incorrect hint pixels can generate severe visual artifacts in colorization.

**Figure 10:**
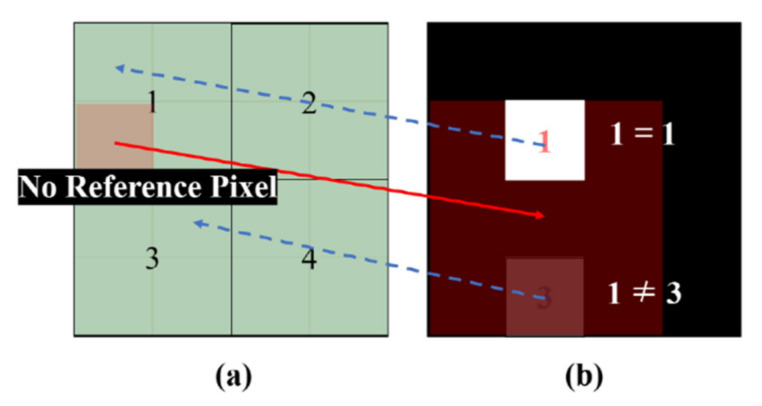
Pruning process for no-reference pixels during color-hint mask generation. (**a**) Color image pixels. (**b**) Color-hint mask associated with the mono image. In (**b**), the color hint with label 3 is removed because the block-based label of the no-reference pixel in (**a**) is different from that label. Here, the blue dashed arrow indicates the disparity compensated position using mono disparity (i.e., from mono to color pixel) and the red arrow indicates the disparity-compensated position using the color disparity (i.e., from color to mono pixel).

A block-based left-right (LR) consistency check is performed at no-reference pixels to remove these possible incorrect color hints. Figure 10 illustrates this pruning process for no-reference pixels. The red square in Figure 10a is a no-reference pixel that is not referred from any pixels in the mono image. At this pixel, disparity compensation is performed while using the color disparity. After that, we check the label of all pixels inside the block centered at the compensated position in Figure 10b with a size of $N_{B1}\times N_{B2}$ pixels (in our experiment, $N_{B2}$ is experimentally set to 5). Block labels that are different indicate that this pixel can be an incorrect color hint, as shown in Figure 10b. Note that this case occurs because of the inconsistency between mono and color disparities. Here, we conservatively prune this type of LR inconsistent pixels from the set of color hints. Therefore, the mask value at the LR incorrect pixel (*x*, *y*) becomes zero (i.e., $M\left(x,y\right)=0$).

### 2.2. Deep Colorization

The most common problem in image colorization is color bleeding artifacts that occur when some dominant color hints cross over the edge in some neighboring regions (see the visual results that are presented in Section 3). Particularly, color bleeding artifacts easily occur in large and complex areas with a few color hints. Because reliable color hints previously obtained are very sparse, the existing colorization methods [12,13] can provide poor results in terms of color bleeding artifacts. Therefore, a new colorization method is required in order to cope with color bleeding artifacts.

Here, we propose a deep learning-based colorization model that mitigates the color bleeding problem. The proposed deep learning model mitigates the color bleeding artifacts by forcing structural information of the mono image into the colorization model. In the remainder of this section, we describe the proposed network architecture, along with the objective function of the network and the extraction of an edge score map for explicit structural information. In addition, the network optimization is described for training the model.

#### 2.2.1. Objective 

The input of the proposed model is $X=\left\{L_{mono},\;\hat{a},\;\hat{b},\;M,\;E\right\}$, where $L_{mono}$ is the luminance channel obtained from the input mono image. $\hat{a}$ and $\hat{b}$ are color-hint values in ab channels of the CIE Lab color space (normalized between −1 and 1). M is the binary color-hint mask, as described in Section 2.1. E is an edge map calculated by the Canny edge algorithm while using the mono image. Here, we used a slightly modified Canny edge algorithm. The Canny edge algorithm originally produces binary values through the double-threshold operation [21]. However, it is not necessary to remove all weak edges (i.e., lower than the high-threshold and bigger than the low-threshold) for our network input. Therefore, in this study, the edge normalization was performed by dividing the weak edge by the high-threshold [21]. Consequently, the final strength of the edge is normalized to a value between 0 and 1.

The proposed deep colorization model ($f:X\to \hat{Y}$) maps a given input *X* to the estimated ab color channels $\hat{Y}\in {\left[-1,1\right]}^{H\times W\times 2}$. In general, to optimize a deep colorization model, *l*1 loss is widely used as pixel-by-pixel loss, because it does not encourage blurring like *l*2 loss. The *l*1 loss is given by:

(7) $$L^{l1}\left(f\right)=E_{X}\left[{\left|Y-f\left(X\right)\right|}_{1}\right],$$

where Y is the ground-truth color version of the input mono image.

It has recently become known that using the structural similarity measure (SSIM) [18] can produce better results [22]. The SSIM computes the structural loss on a local window at each pixel, not just the loss of pixel values. The SSIM loss is given by:

(8) $$L^{SSIM}\left(f\right)=E_{X}[\sum_{p}\left(1-SSIM\left(p;f\left(X\right),\;Y\right)\right)],$$

where *p* is the pixel position and the SSIM is a similarity metric that is generally computed with a window size of 11 × 11 at a pixel position *p* [23]. A recent study [22] has shown that *l*1+SSIM loss can produce better results for image synthesis tasks. In our experiments, we have also observed that *l*1+SSIM loss can produce better results for the colorization model than that of only *l*1 loss (see the Experimental Results section).

The final objective of the model is to find *f* that minimizes the following loss:

(9) $$f^{*}=\underset{f}{argmax}\left(\left(1-\lambda \right)L^{l1}\left(f\right)+\lambda L^{SSIM}\left(f\right)\right),$$

where *λ* controls the amount of influence of *l*1 and SSIM losses, and it was set to 0.84 according to the previous studies [22]. Note that, in [22], 0.84 was found to be optimal for image restoration tasks. We have also observed that the same weight value produces visually plausible results for our experiments.

#### 2.2.2. Network Architecture

**Figure 11:**
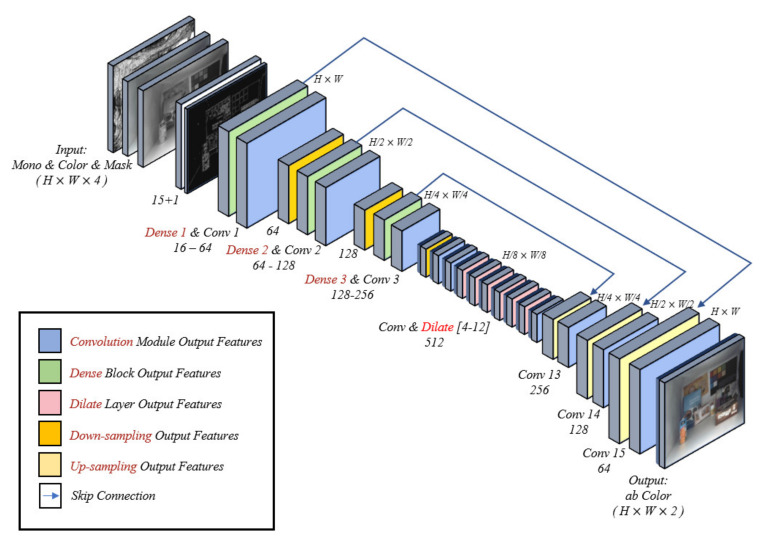
Model architecture of the proposed colorization.

The proposed model is a fully convolutional network using the dense U-net [24,25,26] that takes an input with an arbitrary size and produces the same size output [27], as shown in Figure 11. The dense U-net combines the U-net architecture and dense blocks, as proposed in previous studies [24,28]. The dense block helps to propagate features of the front layers throughout the whole layers of the network [28]. This helps to extract better quality features for colorization tasks. When compared to the previous models for colorization, the proposed model replaces the normal convolution layer in the encoder part of U-net with the dense block.

**Figure 12:**
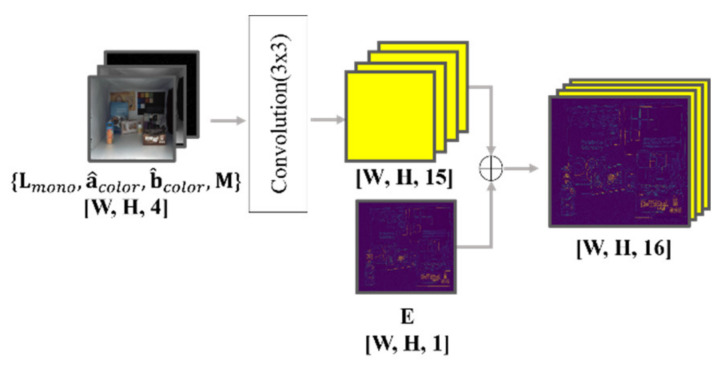
Input layer. The edge information (one channel) is concatenated with the outputs of the first convolutional operations (15 channels).

The input of the proposed model consists of a luminance channel, color hints, and a binary color-hint mask. The input is converted to 15 features through a convolution layer with a 3 × 3 kernel that is learned through training, as shown in Figure 12. The features are then concatenated with the edge information of one channel. It would be helpful if the network were to explicitly know the structural information of the input mono image to prevent color bleeding artifacts because color bleeding artifacts occur mainly around edges in an image. To this end, the proposed model forces edge features of the input image by providing them directly to the network rather than learning them during training. The experimental results reveal that forcing the edge feature into the network can substantially mitigate color bleeding artifacts (see Section 3.2).

The concatenated features are then converted to high-level features in the encoder part. The encoder part consists of three dense blocks, convolution modules with 1 × 1 size kernels (Dense 1–3 and Conv 1–3 in Figure 11), and downsampling layers. After each Dense and Conv operation is performed, each feature tensor is downsampled to half resolution while using the convolution of stride 2. Note that, unlike conventional U-net structures, the number of feature channels is not doubled in downsampling. In the proposed network model, the number of feature channels is determined through dense blocks. However, the number of feature channels is doubled in the last downsampling layer.

**Figure 13:**
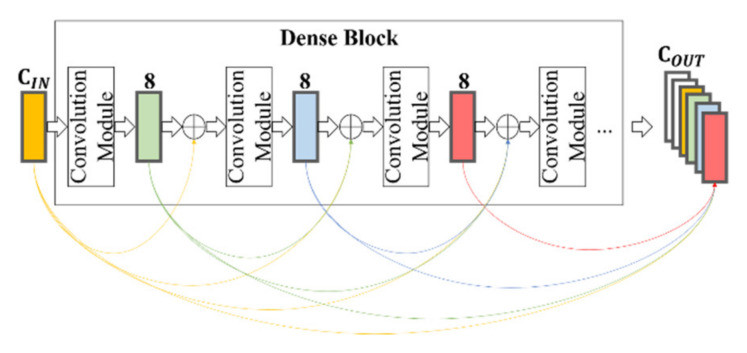
Dense block. Each convolution module forms dense connections by taking all the output features of previous convolution modules as input. Note that, in this example, the growth rate of a dense block is 8. **C_IN_** and **C_OUT_** are the input and output channels of the dense block, respectively.

The aim of using dense blocks is to propagate the edge feature that is provided to the input layer throughout all layers of the network. Consequently, the decoder part of the network can reconstruct visually plausible results without color bleeding artifacts. A dense block consists of several convolution modules composed of BatchNorm-ReLU-Convolution with a 3 × 3 size kernel and stride 1 (in this study, all convolution modules, if not specified, are constructed using the same structure), as shown in Figure 13. The convolution module in the dense block extracts *k* feature channels through the input, where *k* is a hyperparameter that is known as the growth rate. Importantly, the *k* feature channels are concatenated with inputs [28]. The concatenated channels are again fed into the next convolution module. This process is repeated until a specific number of feature channels is reached. In this manner, the dense block forms dense connections between layers, as shown in Figure 13 [28]. Through these dense connections, the resulting feature contains all of the features of each layer of the dense block. In this study, the value of the growth rate *k* is set to 8 and the number of resulting feature channels for the three encoder units is set to 64, 128, and 256, respectively. The output of each dense block is downsampled through a 1 × 1 convolution module.

Convs 4–12 extract features while using the convolution module and dilated convolution module [29] with factor 2 instead of halving the spatial resolution. The dilated filter helps to extract higher-level features without additional down- or upsampling operations, as mentioned in previous studies [29]. The dilated convolution is applied to Convs 6–11.

The decoder part has the same structure as a general U-net. For each Conv in Convs 13–15, the convolution module and upsampling are performed. The feature tensor is spatially doubled while using a 2 × 2 upsampling convolution, and the number of channels is halved. Each upsampling layer has a skip connection from Dense 1–3 blocks. The skip connections help to use the low-level features for reconstructing a final image [30]. In the final layer, estimated ab channels with values between −1 and 1 are obtained through a 1 × 1 convolution layer and hyperbolic tangent activation function.

#### 2.2.3. Network Optimization

The network is optimized by applying the Adam optimizer [31] of a minibatch stochastic gradient descent (SGD) method with a learning rate of 0.0001 and momentum parameters (*β*_1_ = 0.5 and *β*_2_ = 0.999). A random search technique is used for the selection of hyperparameters, as mentioned in previous studies [32]. In the model training stage, the batch size was wet to six and the model was trained for nine epochs, where one epoch consists of 200,000 steps.

The procedures described in Section 2.1 should be performed using stereo image datasets to produce an accurate training dataset for the colorization network. However, no proper stereo image dataset exists to train the colorization network. Furthermore, collecting numerous colorful stereo image dataset is impractical. Instead, we trained using a common image dataset, namely, Places365 [33], almost 1.8 million images with a spatial resolution of 256 × 256 pixels. We have used 1.2 million images from the Places365 dataset to train and the rest of the images from the dataset to validate the model. The mono image, which is the target of the colorization, is obtained through the grayscale conversion function, as used in previous studies [16].

**Figure 14:**
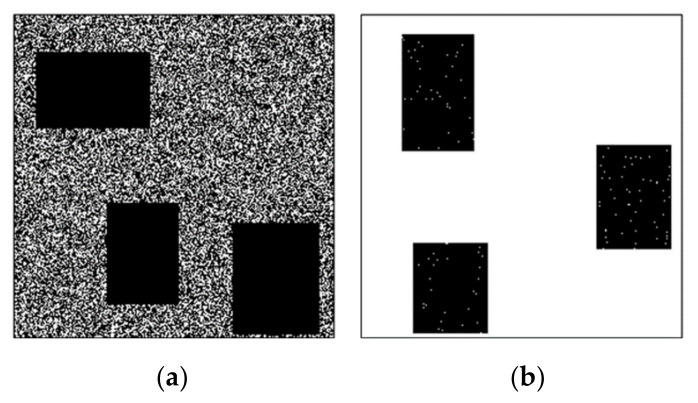
Examples of binary color hint mask. (**a**) Case A. (**b**) Case B. Note that, in the figures, the white pixels (i.e., a value of 1 in the mask) represent hint pixel positions.

The binary hint mask for each image is generated to have the same tendency as the color-hint mask, as described in Section 2.1. The binary color-hint mask generation for training is divided into two cases, A and B. Case A represents a situation that there is no color hint, and it often occurs in occlusion regions (see Figure 4d,f). Therefore, a square hole is used without any hints to simulate such an occlusion region (see Figure 14a). Case B represents a situation that a small number of hints remain in the occlusion region, as observed in the results of the binary color-hint mask generation. A few color hints are added to the square hole to simulate this case (see Figure 14b). Specifically, the mask dataset is constructed, as follows:

Case A. For each ground-truth color image, three rectangular-shaped holes with random sizes of 56–96 pixels are generated at random positions. In addition, random removal is applied for the pixels outside the rectangles at a rate of 0–70%. The remaining pixel positions are used as color hints for the colorization of a given mono image.

Case B. Three rectangular-shaped holes are generated with random sizes of 56–96 pixels at random positions. Subsequently, color hints are given for the random pixels inside the rectangles at a rate of 0–70%.

The random pixel positions outside and inside the rectangles of Cases A and B represent typical sparse color hint patterns that are mainly generated while using the proposed mask generation method (see Figure 4d). Note that the pixels outside the rectangles of Case B are all color hints. This is because we intend for the model to learn that the pixels with color hints must be maintained with the same value in the resulting image. Because Case A mainly occurs in our binary color-hint mask, this case is generated with a 95% probability. The remaining 5% is sampled for Case B.

## 3. Experimental Results

A series of experiments were conducted to verify the performance of the proposed method. Section 3.1 describes that the proposed color transfer method can provide better results than the state-of-the-art methods in terms of image fusion for dual cameras. Section 3.2 describes that the proposed colorization model shows better performance in terms of hint-based colorization than the existing methods.

### 3.1. Comparison of Image Fusion Methods

We conducted experiments to investigate the performance of the proposed color transfer method while using stereo-image datasets consisting of color and mono images. For the experiments, two public datasets were used, namely, the Middlebury dataset [34] and the CVIP LAB stereo dataset [15].

The Middlebury dataset consists of a set of multi-view color images that were captured from multiple RGB cameras for a scene in various illuminance and exposure conditions. For the dual camera simulation, the authors assumed that the left-view was obtained from the color camera and the right-view was obtained from the mono camera. In the case of the Middlebury dataset, two adjacent views were selected in consideration of suitable illuminance for each of the color and mono images. The right-view was simulated as a mono image by converting to grayscale. The grayscale conversion was done according to the Recommendation of ITU-R BT.601-7 [19]. After that, different noises were added to each of the color and mono images to simulate a low-light environment. The parameters used for the simulation are shown in Table 1, and more detailed simulation methods can be found in [4].

The CVIP dataset consists of the color and mono image pairs with a spatial resolution of 1328 × 1048 captured from an RGB + BW camera in various low-light conditions [15]. In the case of the CVIP dataset, the mono camera has the same specifications as the color camera, except that it does not have a color filter array. The mono images were obtained while using a real mono camera and, hence, no simulation was necessary. The detailed camera specifications can be found in the previous study (see Table 1 in [15]). Note that the CVIP dataset is real for low-light environments, but no ground-truth exists. Therefore, the quantitative evaluations were only performed with the Middlebury dataset.

**Figure 15:**
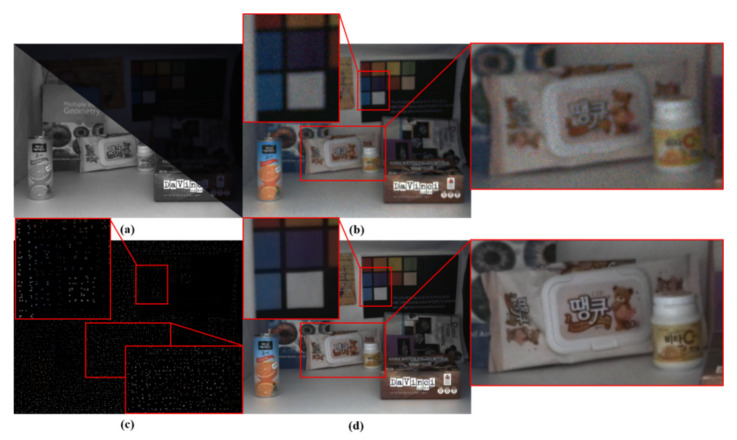
Visual results (6 lux). (**a**) Color- and mono-image pair. (**b**) Histogram matched version of the original color image. (**c**) Binary color-hint mask. (**d**) Fused result by the proposed approach.

Figure 15 shows an example input image provided by CVIP LAB and processing results that were obtained under the six-lux condition. Note that color hints were very sparse (see Figure 15c). Figure 15d shows substantial improvement as compared to the histogram matched version of the original color input image in Figure 15b. In particular, we can observe that the fusion of a color- and mono-image pair yields improved results in terms of denoising and image detail.

In the following experiments, we compare the proposed method with the existing methods. The experiments were conducted while using histogram-matched color- and mono-image pairs. For the quantitative evaluation of the color transfer methods, SSIM [18] and color difference (CIEDE2000) [35] metrics were used. Each value was calculated using the Middlebury dataset. In addition, we show that the proposed model produces visually more plausible results than the existing methods while using the Middlebury and CVIP LAB datasets.

#### 3.1.1. Comparison with Color Transfer Methods

First, we compared the proposed method with the existing color transfer methods, i.e. Welsh [7], Irony [8], Gupta [9], Jeon [4], He [10,11], Dong [5,6], and Zhang [13]. For a fair comparison, the search range of Welsh’s [7], Irony’s [8] and Gupta’s [9] methods were adjusted to each scanline of a given stereo image pair. In Zhang’s method [13], the color hints were given by using the proposed color hint mask generation.

**Figure 16:**
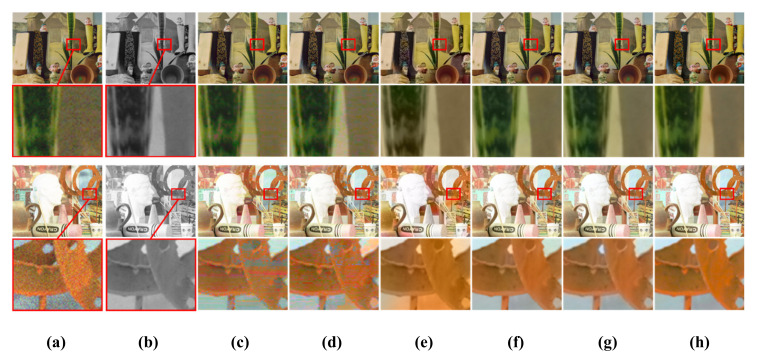
Visual comparison for various non-deep learning-based color transfer methods with Middlebury stereo dataset. (**a**) Histogram-matched version of the original color image. (**b**) Monochrome image. Results from (**c**) Welsh [7], (**d**) Irony [8], (**e**) Gupta [9], (**f**) Jeon [4], (**g**) proposed, and (**h**) ground truth. The first row shows the input and output from the setup 1 condition of Table 1. The second row shows the close-up images of the red area of the first row. The third row shows the input and output from the setup 2 condition of Table 1. The fourth row shows the close-up images.

**Figure 17:**
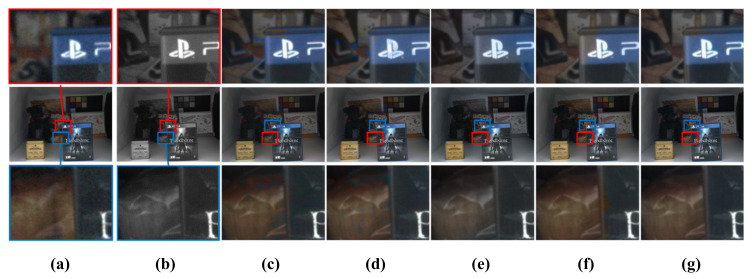
Visual comparison for various non-deep learning-based color transfer methods with a dual-camera dataset (10 lux). (**a**) Histogram-matched version of the original color image. (**b**) Mono image. Results from (**c**) Welsh [7], (**d**) Irony [8], (**e**) Gupta [9], (**f**) Jeon [4], and (**g**) proposed. The middle row shows the input and outputs. The first row and the third show the close-up images.

Table 2 shows the quantitative results of the color transfer methods in terms of SSIM, CIEDE2000, and CPSNR. Note that the SSIM metric measures how well the structural information of a resulting image is reconstructed [18]. In our experiment, the SSIM value was calculated in a channel-wise manner at 11 × 11 window around reconstructed color pixels, except for color hints. In other words, it is calculated as the average of the three SSIM values calculated per each channel of an RGB image. In addition, the CIEDE2000 metric was used to measure the degree of difference between the two colors of the ground-truth and reconstructed images [35]. In addition, the color peak signal to noise ratio (CPSNR) calculates PSNR in a channel-wise manner and it takes an average PSNR for the image quality assessment of color images [36]. The quantitative evaluation results show that the proposed method outperforms the existing color transfer methods, as shown in Table 2. Figure 16 and Figure 17 show the visual results of non-deep learning-based color transfer methods for the Middlebury and CVIP LAB datasets, respectively. Welsh’s and Irony’s methods [7,8] resulted in serious visual artifacts, because they did not consider luminance differences between color and mono images and noises that often occur in low-light conditions, as shown in Figure 16c,d and Figure 17c,d. As shown in Figure 16e and Figure 17e, Gupta’s method [9] shows less colorful results because the stereo matching is performed while using superpixels. In addition, color bleeding artifacts occurred. Jeon’s method [4] showed good performance in terms of SSIM, CIEDE2000, and CPSNR, because they addressed the color bleeding artifact while using a local color consistency map, which assigns low weights to incorrect color hints caused by disparity error. However, we observed that it cannot deal with all the incorrect hints that often occur in large occlusion regions, as shown in Figure 17f. When compared with Jeon’s method [4], the proposed method does not propagate incorrect color hints in large occlusion regions because the proposed method aggressively removes possible incorrect color hints by the color-hint generation algorithm that is described in Section 2.1.

**Figure 18:**
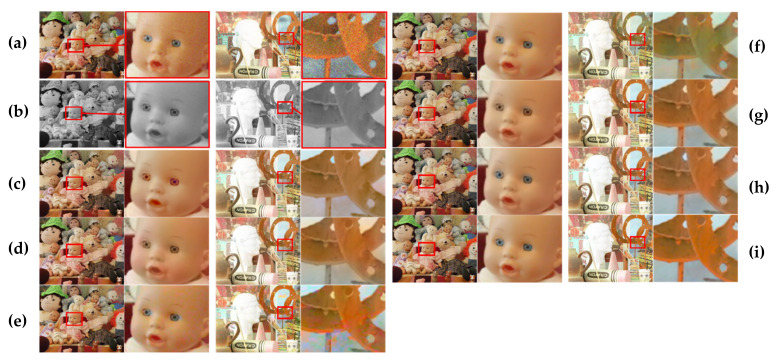
Visual comparison for various deep learning-based color transfer methods with Middlebury stereo dataset. (**a**) Histogram-matched version of the original color image. (**b**) Mono image. Results from (**c**) He [10], (**d**) He [11], (**e**) Dong [5], (**f**) Dong [6], (**g**) Zhang [13], (**h**) proposed, and (**i**) ground truth. The first column shows the input and output from the setup 1 condition of Table 1. The second column shows the close-up images of the red area of the first column. The third column shows the input and output from setup 2 condition of Table 1. The fourth column shows the close-up images of the red area of the third column.

**Figure 19:**
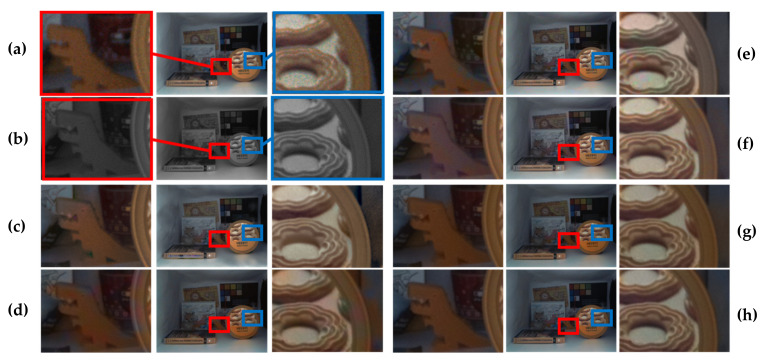
Visual comparison for deep learning-based color transfer methods with a dual-camera dataset (10 lux). (**a**) Histogram-matched version of the original color image. (**b**) Mono image. Results from (**c**) He [10], (**d**) He [11], (**e**) Dong [5], (**f**) Dong [6], (**g**) Zhang [13], and (**h**) proposed. The middle column shows the input and output. The first column and the third column show the close-up images of the red area and blue area of the middle column.

Figure 18 and Figure 19 show the visual results of the learning-based color transfer methods for the Middlebury and CVIP LAB datasets, respectively. The results of He’s methods [10,11] were colorized with different colors from the ground-truth (see Figure 18c–d for the eyes of the doll), because the purpose of their methods is to minimize semantic differences in unnatural colorization. In addition, it was observed that the matching for hints fails when we apply it for the stereo image pairs captured in the real low-light environment (see Figure 19c–d). In the case of Dong’s method [5], we observed that the matching fails, because the assumptions on gray-color correspondence prior are not true in the low-light environment (see Figure 19e). In addition, this method was not able to handle noises in low-light environment. In a recent study [6], Dong et al. also proposed a method that effectively solves the denoising problem through attention operations. However, in some cases, the mismatching often occurred because of intensity differences between the color and mono images captured in low-light conditions. Particularly, this mismatching issue severely occurred in Setup 2, where the difference in light conditions of color and mono images was large (see Figure 18f). Zhang’s method [13] showed good results in terms of SSIM and CIEDE2000. This is because the color hint mask generation method that is proposed in this paper removes incorrect color hints that may occur in occlusion areas. However, we observed that the dominant color hints invaded the object’s boundary and, hence, caused color bleeding artifacts, as shown in Figure 19g.

#### 3.1.2. Comparison with Detail Transfer Method

**Figure 20:**
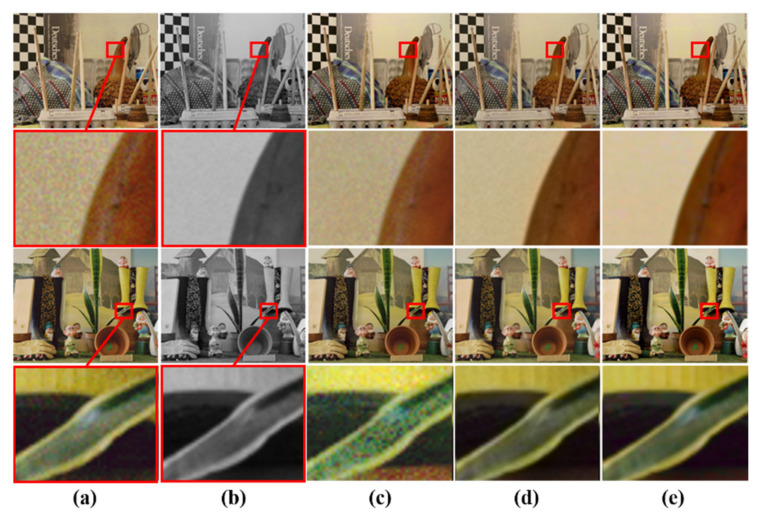
Middlebury dual-camera dataset comparison with detail transfer method. (**a**) Histogram-matched version of the original color image. (**b**) Mono image. (**c**) Results from Jung [15]. (**d**) Proposed. (**e**) Ground truth. The first and third rows show the input and output from the setup 1 condition of Table 1. The second and fourth rows show the close-up images of the first and third rows, respectively.

**Figure 21:**
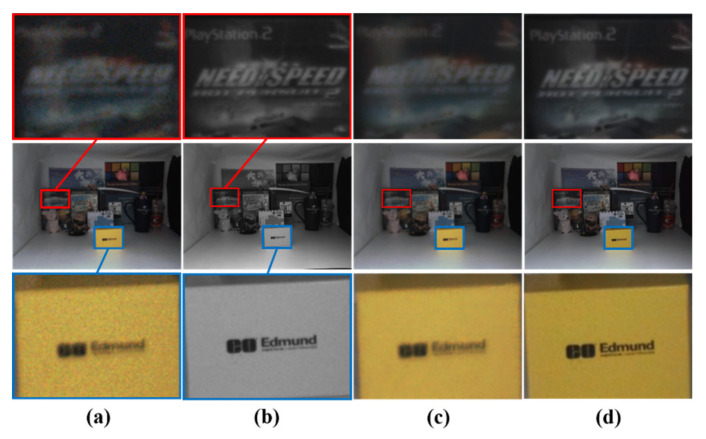
Dual-camera dataset comparison with detail transfer (6 lux). (**a**) Histogram-matched version of the original color image. (**b**) Mono image. (**c**) Results from Jung [15]. (**d**) Proposed. The middle row shows the input and output. The first row and third row show close-up images of the red area and the blue area of the middle row, respectively.

We also compared the proposed methods with the state-of-the-art detail transfer (i.e., Jung [15]). Table 3 shows the comparison results with the detail transfer method that transfers the detail information of the mono image to the color image. The experimental results show that the proposed method can achieve better performance than that of Jung’s method in terms of SSIM (the structural similarity). This is natural, because the proposed color transfer method fully maintains the detailed information of the mono image. Note that Jung’s method [15] attempts to solve disparity problems by selectively transferring the detail information from the mono to the color image, depending on the degree of disparity error. However, detail loss inevitably occurs, as shown in Figure 20c and Figure 21c. When compared with Jung’s method [15], the proposed method does not cause the loss of detail, because the mono channel remains unchanged as part of the original input.

Moreover, the performance in terms of the color difference measure is similar to Jung’s method, as seen in Table 3 [15]. Note that the CIEDE2000 result of the proposed method is not higher than 2.3 (i.e., the just noticeable color difference (JNCD) [35]). This means that humans do not perceive color artifacts in the final images. Overall, these results indicate that the proposed method outperforms the existing detail transfer method.

### 3.2. Colorization Network Analysis

We conducted further experiments in order to investigate the performance of the proposed deep colorization network. First, we compared the proposed colorization network with the existing hint-based colorization methods for the analysis of colorization performance. We then analyzed the effect of edges on color bleeding and the effect of color hint mask. 

#### 3.2.1. Comparison with Hint-based Colorization Methods

The performances of the existing hint-based colorization methods were evaluated for comparison. The comparative analysis must be performed with hint-based colorization methods using color hints because the purpose of this study is not automatic colorization without any color hints. For the comparison, we used Levin’s colorization [12] and Zhang’s colorization models [13], which are user-hint-based colorization methods.

Levin’s colorization [12] is a conventional least-square optimization-based colorization method. We used the program codes of Levin colorization provided on a public website for comparison. Zhang’s colorization [13] is a deep learning-based colorization model. It was designed for both user-hint-based and automatic colorization. We retrained Zhang’s colorization model while maintaining the original architecture because it is unfair to compare it with the proposed model trained for only cases with color hints [13]. Specifically, *l*1 loss was used, as proposed in the original study [13]. In addition, for a fair comparison, *l*1+SSIM loss was used for retraining as used in the proposed method. Retraining was conducted in the same manner as the proposed model.

To summarize, the following four hint-based colorization methods were used to compare colorization performances: (1) Levin’s colorization [12], (2) Zhang’s colorization model with *l*1 loss [13], (3) Zhang’s colorization model with *l*1+SSIM loss [13], and (4) the proposed model. The evaluation to compare the performance of hint-based colorization methods was designed, as follows. For visual comparison, a set of diverse colorful test images of 1280 × 960 pixels were collected from an image download site [37,38]. The color holes were randomly generated (with random sizes and random positions) in ab channels for each test image. The remaining color values were used as color hints for the four colorization methods.

**Figure 22:**
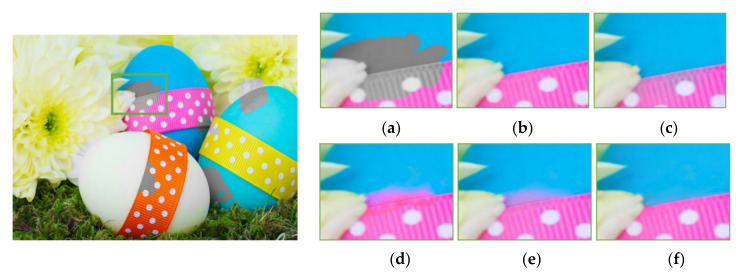
Visual comparison results of colorization performance. (**a**) Input image with a color hole. (**b**) Ground truth. (**c**) Levin colorization [12]. (**d**) Zhang model (*l*1 loss) [13]. (**e**) Zhang model (*l*1 + SSIM loss) [13]. (**f**) Proposed model.

**Figure 23:**
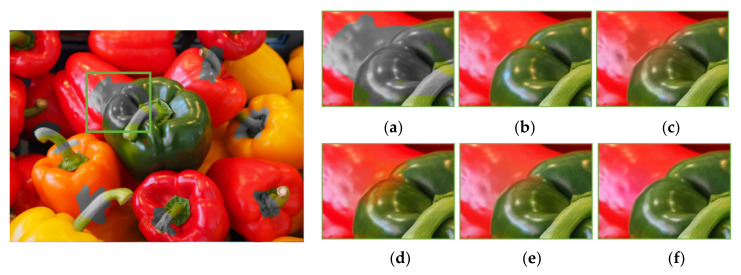
Visual comparison results of colorization performance. (**a**) Input image with a color hole. (**b**) Ground truth. (**c**) Levin colorization [12]. (**d**) Zhang model (*l*1 loss) [13]. (**e**) Zhang model (*l*1 + *SSIM* loss) [13]. (**f**) Proposed model.

As previously mentioned, the existing methods of color hint-based colorization suffer from color bleeding artifact problems around object boundaries with few color hints. Some color (blue or pink) in a region spilled over its neighbors, as shown in Figure 22c–e and Figure 23c–e. It caused severe visual artifacts in the resulting image. Figure 22 and Figure 23 show more visual comparison colorization results. These results demonstrate that the proposed deep learning-based colorization method yielded improved visual results as compared to the existing methods.

The SSIM [18] and color difference (CIEDE2000) metric [35] were also used for the quantitative evaluation of the colorization methods. Each value was calculated while using a test image set. Specifically, the test set consisted of 100 images that were randomly collected from the Places365 dataset [33] with a spatial resolution of 256 × 256 pixels. In this experiment, the binary color-hint mask for each image was generated by making a square hole with a size ranging from 32 to 160 pixels at random positions. Note that in this experiment, we investigated the colorization performance for color holes with various sizes, because occlusion holes usually generate color bleeding artifacts in our application (i.e., image fusion for a dual camera).

Table 4 shows the results of the SSIM metric. The proposed colorization method outperformed the existing methods for all cases of hole size, thus demonstrating that the proposed colorization method can achieve better results than the existing colorization methods in terms of the SSIM quality measure, as shown in Table 4. Recall that the proposed method forces the structural information into the deep-learning model, and thus the colorized output image can reconstruct better structural information than the existing methods. The evaluation results that were obtained by SSIM clearly support the advantage of the proposed model.

Table 5 shows the results of the calculations of the CIEDE2000 formula. The proposed model reconstructed the color with the least difference from the ground-truth images as compared to the other methods, as shown in Table 5. This suggests that the proposed model can reconstruct colors that are more similar to those of the ground-truth images than those of the existing methods.

#### 3.2.2. Effect of Edge on Color Bleeding

The visual results of Section 3.2.1 show that the proposed colorization model can achieve better results in terms of color bleeding artifacts. Furthermore, an experiment was conducted in order to investigate whether the use of pre-calculated edge features actually affect the mitigation of color bleeding artifacts. To this end, two networks were used. One had the same structure as the proposed model. The other network removed the edge feature and adjusted the convolution output channel of the first layer to 16. They were trained in the same environment, except that the edge feature was excluded.

**Figure 24:**
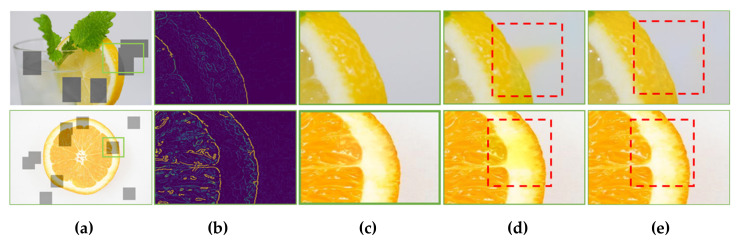
Effects of edge on color bleeding artifacts. (**a**) Color and mono input with color hint. (**b**) Edge score map. (**c**) Ground-truth. (**d**) Proposed model with no edge information. (**e**) Proposed model with edge input. In (**e**), the use of edge information mitigates erroneous color propagation in a large hole.

Figure 24 shows the comparison results of the influence of the edge feature. In the model without the edge feature, the dominant color hint invaded the edge in some neighboring regions (see Figure 24d). However, it is clearly observed that color bleeding artifacts did not occur in the proposed model provided with the edge feature as an input. Figure 24e shows that the color bleeding artifacts were mitigated in the proposed colorization model with the edge feature.

#### 3.2.3. Effect of Color Hint Mask

We compared the performance of the colorization network with and without the color hint mask as input for the ablation study of the proposed method. A deep colorization model was trained without the color hint mask to analyze the influence of the binary color hint mask. That is, the color hint mask was not given as a network input. It can be seen from Table 6 that the proposed model with the color hint mask outperforms the model without the color hint mask. This is because the deep model without color hint mask cannot handle all of the incorrect color hints that occurred from inaccurate per-pixel registration (e.g., in occluded regions).

### 3.3. Discussion

This study proposes a novel color transfer method using a deep-learning-based colorization process with reliable color hints, which can mitigate the color bleeding and false color artifacts. In the experiments, the proposed method is compared with the existing methods through various image datasets (i.e. Middlebury and CVIP datasets). The experimental results show that the proposed method outperforms the state-of-the-art methods for image fusion of color- and mono-image pairs.

We provide additional information regarding the tunable parameters (i.e., block size $N_{B1}$ and $N_{B2}$ and reliability parameter β) in the proposed color-hint mask generation process. Table 7 shows the SSIM results that were obtained by different sets of $N_{B1}$ and $N_{B2}$. $N_{B}$ controls the amount of color hints, as described in Section 2.1.2 and Section 2.1.3. If $N_{B}$ increases, the number of color hints decreases. This can further eliminate incorrect color hints that are caused by per-pixel registration, but the number of color hints for color restoration is also reduced. Conversely, as $N_{B}$ decreases, the number of color hints increases. This increases the number of color hints required for colorization, but the number of incorrect color hints also increases. A grid search technique was used with a set of block sizes in order to find the optimal values of parameters $N_{B1}$ and $N_{B2}$. In addition, the parameter β controls the influence of BJND. As beta approaches 0, the reliability value becomes equal to the similarity value of image patches (i.e., Equation (3)). Conversely, as the value of the parameter increases, the reliability is dominantly determined by BJND. This means that a high-reliability value is given to the area where the human visual system is difficult to detect the color difference. In our experiments, the optimal value was determined to be 1.5 through a grid search.

In this study, the CIE Lab color space was adopted for deep colorization. One of the major reasons is that CIE Lab color space can separate the chrominance from the luminance in an image. Additionally, the color distance in the CIE Lab color space is more suitable for the colorization tasks than other correlated color spaces since it is based on human color perception [13]. Note that the previous state-of-the-art method for deep learning-based colorization [13] also used the CIE Lab color model. The proposed method is also applicable to the other correlated uniform color spaces (e.g., Luv) for the same reasons. To investigate this, an additional experiment was conducted by training the proposed network while using the Luv color space. However, the results of the Luv model do not differ significantly from that of the Lab model (0.9738 of SSIM and 2.12 of CIEDE2000 for the Luv model vs. 0.9737 and 1.99 for the Lab model).

It should be further noted that the TV-L1 optical flow method was adopted for stereo matching [17]. Note that this optical flow method has been also used for many previous studies (e.g., disparity remapping [39,40]) and publicly available to the code. The code is originally implemented in MATLAB, and it takes 180.2 s for an image with a resolution of 640 × 480 in an Intel Core i5-8600 CPU @ 3.10GHz. Note that the CPU processed the code. However, using GPU can significantly reduce the TV-L1 optical flow processing time (180.2 s in CPU vs. 1/30 s for real-time operation in GPU), as mentioned in previous studies [17].

Furthermore, note that the other disparity estimation algorithms can be also used instead of the TV-L1 optical flow in the proposed method. To investigate this, an additional experiment was conducted in order to analyze the effects of a different stereo matching algorithm. For the experiment, the Semi Global Block Matching (SGBM) [41] algorithm was adopted, which is one of the most well-known stereo matching algorithms and it is treated as the baseline performance for non-deep learning algorithms. The final fusion results obtained by the SGBM algorithm also outperform the other existing methods (for the SSIM, 0.9633 of SGBM vs. 0.9737 of TV-L1). This result supports that the proposed color-hint mask generation can remove incorrect color hints effectively, even if the stereo matching algorithm is changed.

We compared the computational cost of the proposed network with the previous network. Note that the proposed network is based on hint-based colorization. Therefore, Zhang’s method [13], the state-of-the-art of the hint-based colorization, was selected for the comparison. In general, the number of learnable parameters and floating-point operations (FLOPs) calculate the computational cost of a deep learning model [42]. We used the profile function that was provided by the TensorFlow library to calculate the FLOPs of the network. FLOPs were calculated only for trainable parameters to generalize the calculation of FLOPs. The number of floating-point operations is 112.26 GFLOPs for Zhang’s method [13] vs. 75.92 GFLOPs for the proposed method. The number of learnable parameters is 33.34 M for Zhang’s method vs. 25.31 M for the proposed method. Note that both the number of learnable parameters and FLOPs are less than the previous network. This is due to the advantages of the dense blocks used in our network architecture. The dense blocks can perform better in terms of memory efficiency and FLOPs, as mentioned in the previous study [27].

## 4. Conclusions

This study proposed a deep color transfer method that enhances low-light images captured by a color-plus-mono dual camera. The proposed algorithm conservatively prunes incorrect color hints to solve the visual artifact problems that are caused by disparity errors. In addition, image colorization is performed through a deep colorization network using the correct color hints and edge features to mitigate the color bleeding artifacts. Experimental results revealed that the proposed algorithm is robust to color bleeding artifacts and large disparity errors. In addition, the comparison results showed that the proposed method outperforms the state-of-the-art methods in terms of the image colorization performance and, thus, image fusion. These experimental results demonstrate that the deep color transfer method can provide significant improvement in terms of low-light-level performance using a color-plus-mono dual camera. Future work will develop optimized models that can be used in small devices (e.g., embedded devices and smartphones).

## Figures and Tables

**Table 1:** Experimental setup conditions for dataset construction.

	Color Camera			Monochrome Camera		
	Illum.	Exp.	Noise Std.	Illum.	Exp.	Noise Std.
Setup 1	1	0	$0.03^{\sqrt{k}}$	2	1	$0.01^{\sqrt{k}}$
Setup 2	3	0	$0.07^{\sqrt{k}}$	1	2	$0.01^{\sqrt{k}}$

**Table 2:** Quantitative evaluation results with the existing color transfer methods in different simulation setups.

	Setup 1			Setup 2		
	SSIM	CIEDE2000	CPSNR	SSIM	CIEDE2000	CPSNR
Welsh et al. [7]	0.8738	6.08	26.68	0.8773	9.37	24.11
Ironi et al. [8]	0.8945	6.88	27.70	0.8940	8.33	23.14
Gupta et al. [9]	0.8491	6.28	25.53	0.8851	8.91	22.58
Jeon et al. [4]	0.9590	2.47	31.72	0.9466	3.88	28.51
He et al. [10]	0.8920	5.63	27.61	0.8911	5.77	26.04
He et al. [11]	0.9042	5.43	28.96	0.9104	5.43	27.22
Dong et al. [5]	0.9201	4.52	29.47	0.9120	6.12	24.30
Dong et al. [6]	0.9574	2.83	31.66	0.9167	5.80	26.72
Zhang et al. [13]	0.9589	2.55	31.72	0.9501	4.00	26.76
Ours	0.9737	1.99	32.10	0.9642	3.20	29.25

**Table 3:** Structural similarity measure (SSIM) and CIEDE 2000 metric results for the comparison with the detail transfer method.

	SSIM	CIEDE2000
Jung [15]	0.9501	1.86
Ours	0.9737	1.99

**Table 4:** SSIM results for quantitative evaluation.

Square-Hole Size (Pixels)	Levin [12]	Zhang (*l*1 Loss) [13]	Zhang (*l*1+*SSIM* Loss) [13]	Proposed
32 × 32	0.9695	0.9726	0.9758	0.9791
64 × 64	0.9501	0.9591	0.9647	0.9688
96 × 96	0.9341	0.9511	0.9552	0.9611
128 × 128	0.9126	0.9398	0.9452	0.9516
160 × 160	0.8984	0.9233	0.9292	0.9418

**Table 5:** Color difference results based on the CIEDE2000 metric.

Square-Hole Size (Pixels)	Levin [12]	Zhang (*l*1 Loss) [13]	Zhang (*l*1+*SSIM* Loss) [13]	Proposed
32 × 32	3.10	2.80	2.48	2.43
64 × 64	4.05	3.52	3.23	3.10
96 × 96	4.78	4.12	3.87	3.61
128 × 128	5.53	4.51	4.26	3.98
160 × 160	6.01	5.07	4.72	4.33

**Table 6:** Ablation study on effects of color hint mask in the proposed colorization network.

	SSIM	CIEDE2000
Without the color hint mask	0.9334	3.44
With the color hint mask	0.9737	1.99

**Table 7:** Selection of the block size parameters (in terms of SSIM).

		$N_{B2}$		
		3	5	7
$N_{B1}$	7	0.9649	0.9650	0.9648
	9	0.9736	0.9737	0.9731
	11	0.9701	0.9701	0.9621
